# ROS-mediated iron overload injures the hematopoiesis of bone marrow by damaging hematopoietic stem/progenitor cells in mice

**DOI:** 10.1038/srep10181

**Published:** 2015-05-13

**Authors:** Xiao Chai, Deguan Li, Xiaoli Cao, Yuchen Zhang, Juan Mu, Wenyi Lu, Xia Xiao, Chengcheng Li, Juanxia Meng, Jie Chen, Qing Li, Jishi Wang, Aimin Meng, Mingfeng Zhao

**Affiliations:** 1 Department of Hematology, Tianjin First Central Hospital, Tianjin 300192, China; 2 Tianjin Key Lab of Radiation Medicine and Molecular Nuclear Medicine, Institute of Radiation Medicine, Academy of Medical Science and Peking Union Medical College, Tianjin 300192, China; 3 Department of Hematology, The Affiliated Hospital of Guizhou Medical University, Guiyang 550004, China

## Abstract

Iron overload, caused by hereditary hemochromatosis or repeated blood transfusions in some diseases, such as beta thalassemia, bone marrow failure and myelodysplastic syndrome, can significantly induce injured bone marrow (BM) function as well as parenchyma organ dysfunctions. However, the effect of iron overload and its mechanism remain elusive. In this study, we investigated the effects of iron overload on the hematopoietic stem and progenitor cells (HSPCs) from a mouse model. Our results showed that iron overload markedly decreased the ratio and clonogenic function of murine HSPCs by the elevation of reactive oxygen species (ROS). This finding is supported by the results of NAC or DFX treatment, which reduced ROS level by inhibiting NOX4 and p38MAPK and improved the long-term and multi-lineage engrafment of iron overload HSCs after transplantation. Therefore, all of these data demonstrate that iron overload injures the hematopoiesis of BM by enhancing ROS through NOX4 and p38MAPK. This will be helpful for the treatment of iron overload in patients with hematopoietic dysfunction.

A substantial proportion of patients with primary or secondary bone marrow failure syndromes, such as aplastic anemia (AA), myelodysplastic syndromes (MDS), myelofibrosis (MF) or β-thalassemia, require frequent transfusions of suspended red blood cells (RBCs). A unit of red blood cells contains approximately 100 mg of iron, but there is a lack of a physiological mechanism for iron excretion^1^,^2^. Consequently, these patients develop transfusion-dependent iron overload. Many studies have demonstrated that excess iron released from aging and damaged erythrocytes could deposit in the parenchymal organs, including the liver, heart, pancreas, brain and joints; ionic iron-mediated toxicity in these organs could enhance the effects of oxidative stress and ultimately lead to the dysfunction of visceral organs, such as congestive heart failure, arrhythmias, cirrhosis, hepatocellular carcinoma, insulin resistance and diabetes, arthritis, fatigue and sexual dysfunction^3^,^4^,^5^.

Although iron overload has a clear effect onparenchyma organs, the effects of iron overload on the hematopoietic system have not been elucidated, and the exact mechanism is uncertain. Increasing clinical evidence has indicated that iron overload has a suppressive effect on hematopoiesis in MDS or AA patients and that iron chelation therapy could improve this situation^6^,^7^. Since it is difficult to investigate the exact mechanism in these patients on the basis of limited human samples and individual differences, there is no relevant reports on this mechanism. Therefore, it is necessary to establish iron overload models to stimulate clinical situations.

Iron-overloaded cell models of bone marrow mononuclear cells (BMMNCs) and mesenchymal stem cells (MSCs) were established in our preliminary studies^8^. Iron overload could impair hematopoiesis by damaging hematopoietic cells and the hematopoietic microenvironment, which is mediated by reactive oxygen species (ROS)-related signaling proteins *in vitro*. Although these findings may partly explain how iron overload affects hematopoiesis, it remains unclear whether iron overload impairs hematopoietic function by enhancing oxidative stress *in vivo*.

In this paper, we first confirmed that the hematopoietic inhibitory effects of iron overload in an iron-overloaded mouse model were parallel to clinical conditions. Secondly, its related mechanism was investigated. It was demonstrated that iron overload increased the ROS levels of HSPCs through the NOX4/ROS/P38 MAPK signaling pathways. This information is useful for further studies on this mechanism and would provide an experimental basis for a new therapeutic target in the treatment of iron overload in patients with hematopoietic dysfunction.

## Materials and methods

### Ethics Statement

The study was approved by the Institutional Animal Care and Use Committee of PUMC and the methods were carried out in accordance with the approved guidelines.

### Reagents

Anti-mouse-CD45.1-FITC, CD45.2-PE, Gr-1-PE/CY7, CD45R/B220-PE/CY5.5 CD11b-PE/CY7 and CD3-APCwere purchased from BioLegend (San Diego, CA, USA); anti-mouse-Sca-1-PE, CD117 (c-kit) Alexa Fluor 700, CD4, CD8, CD45R/B220, Gr-1, CD11b, Ter119 and APC/CY7-conjugated streptavidin were purchased from eBioscience (San Diego, CA, USA); iron-dextrin was purchased from Pharmacosmos A/S (Denmark); the ROS staining kit (S0033) and NAC were purchased from the Beyotime Institute of Biotechnology; calcein-AM fluorescent dye was purchased from Sigma-Aldrich (USA); deferasirox was purchased from Novartis; RPMI 1640 was purchased from Gibco (USA); methylcellulose M3434 was purchased from Stem cell (USA); fetal calf serum was purchased from Bioind (Italy); CD117 MicroBeads (130-091-229) were purchased from Miltenyi Biotec (Germany); a RNA PCR Kit (AMV) Ver.3.0 (DRR019A) was purchased from Takara; a RNeasy MicroKit (74004) was purchased from Qiagen (Germany); the NOX4 and GPX1 gene qRT-PCR primers were synthesized by Sangon Biotech (Shanghai, China).

### Animals and treatments

Male C57BL/6-Ly-5.1 (Ly45.1) and C57BL/6-Ly5.2 (Ly45.2) mice were purchased from the Institute of Laboratory Animal Sciences (PUMC, Beijing, China) and from Vital River (Beijing, China). The Ly45.1/45.2 mice were bred at the certified animal care facility in the Institute of Radiation Medicine of PUMC. The mice were housed with 3-5 individuals per cage and were used at a weight of approximately 20.0-25.0 g. The Ly45.2 mice were the experimental mice, Thirty-six male mice (Ly45.1) were the recipient mice and Eight Ly45.1/45.2 mice were the competitive mice. Forty male mice (Ly45.2) were randomly divided into four groups: (a) a control group (CTL); (b) a low-dose iron group (12.5 mg/ml); (c) a medium-dose iron group (25 mg/ml); and (d) a high-dose iron group (50 mg/ml). The control group was injected with normal saline and the iron overload groups were injected with different doses of iron dextran intraperitoneally (0.2 ml) every three days for four weeks. The deposition of iron in the liver, spleen and bone marrow were assessed using hematoxylin and eosin (HE) staining and Perls’ iron staining. Twenty male mice (Ly45.2) were randomly divided into four groups: (a) a CTL group; (b) an iron overload (IO) group (25 mg/ml); (c) an IO + NAC group; and (d) an IO + DFX group. The IO + NAC group mice were given NAC in drinking water (40 mM). The water bottles were changed twice per week with a freshly made NAC solution. The IO + DFX group mice received 2.5 mg DFX via gavage twice every three days for four weeks.

### Peripheral blood cell and BM mononuclear cell (BMMNC) counts

We obtained the peripheral blood from anesthetized mice via the orbital sinus and collected the blood samples in ethylenediaminetetraacetic acid (K_3_EDTA) tubes. Complete blood counts were obtained using a pocH-100i hematology analyzer (Sysmex, Japan). The cell counts included white blood cells (WBCs), the percentages of neutrophils (NE%) and lymphocytes (LY%), red blood cells (RBCs), hemoglobin (HGB) and platelets (PLTs). The BMMNCs were flushed from the bones as described previously^9^,^10^ and were counted using the hematology analyzer.

### Flow cytometric assays

The BMMNCs were stained with PE-conjugated anti-Ter-119 or the biotin-conjugated antibodies Gr-1 and CD11b; the streptavidin APC-CY7 was incubated with DCFH-DA (10 μM) or calcein-AM (0.125 μM) in a humidified atmosphere of 5% CO_2_ in air at 37°C for 15 min. The hematopoietic progenitor cells (HPCs) (Lin^–^c-kit^+^Sca-1^−^), hematopoietic stem cells (HSCs) (Lin^–^c-kit^+^Sca-1^+^) and long-term hematopoietic stem cells (LT-HSCs) (CD34^–^Lin^–^c-kit^+^Sca-1^+^) were analyzed as described previously^10^,^11^, and the levels of intracellular ROS and labile iron pool (LIP) were analyzed by measuring the mean fluorescence intensity (MFI) of 2’-7’dichlorofluorescein (DCF) or calcein using a flow cytometer.

### Colony-forming cell (CFC) assay

CFC assays were performed by culturing BMMNCs in MethoCult GF M3434 methylcellulose medium (Stem Cell Technologies, Vancouver, BC). Colony-forming unit granulocyte-macrophage (CFU-GM), colony-forming unit erythroid (CFU-E), burst-forming unit erythroid (BFU-E) and colony-forming unit mix (CFU-Mix) were counted on days 5, 7, 9 and 12, respectively, using a microscope according to the manufacturer’s protocol.

### Competitive repopulation assay (CRA)

Competitive repopulation assays were performed using the Ly45 congenic mice to analyze hematopoietic stem reconstitution capacity, as described previously^10^. Donor BMMNCs were harvested from the Ly45.2 mice after they were given different treatments. These cells (1 × 10^6^ BMMNCs) were mixed with competitive cells (1 × 10^6^ BMMNCs) from the Ly45.1/45.2 hybrid mice. The mixed cells were transplanted into lethally irradiated (4.5 Gy twice) Ly45.1 recipient mice (ten mice/group) via lateral canthus vein injection. Peripheral blood was obtained from all of the recipients at two months and four months after transplantation and was analyzed using a BD FACS Aria III, as described previously^10^. Furthermore, secondary transplatation had also been done as above.

### Single-cell colony assay

Sorted CD34^-^Lin^-^sca1^+^c-kit^+^cells (CD34^-^LSK^+^cells) were seeded into the wells of 96-well round-bottom micro plates using theBD FACS Aria III cell sorter at a density of 1 cell/well. The cells were cultured in 200 ml IMDM supplemented with 10% fetal calf serum, 1% bovine serum albumin, 2 mM L-glutamine, 50 mM 2-b-mercaptoethanol, and 10 ng/ml stem cell factor, 10 ng/ml thrombopoietin, and 10 ng/ml IL-3, as described previously. After 14 days of culture, the colonies of cells with ≥50 cells/well were scored under an inverted microscope. The results are expressed as the number of colonies per 20 wells.

### Quantitative real-time assay

We extracted the total RNA from the sorted HPCs and HSCs using the TRizol reagent (Life Technologies, Grand Island, NY, USA) followingthemanufacturer’sprotocol. The cDNA Samples were mixed with primers and the SYBR Master Mix (Life Technologies) in a total volume of 25 ml. All of the samples were analyzed in triplicate using an ABI Prism 7500 sequence detection system. The threshold cycle (CT) values for each reaction were determined and averaged using TaqMan SDS analysis software (Applied Biosystems, Life Technologies). The changes in the expression of a target gene were calculated using the comparative C_T_ method (fold changes = 2^−ΔΔCT^), as described previously.

### Western blotting

The total proteins were obtained using protein isolation kits (Beyotime Institute of Biotechnology) based on the manufacturer’s protocol. The protein extracts were subjected to SDS-PAGE and then transferred to PVDF membranes. After blocking in bovine serum albumin (BSA) for 1 h, the proteins were probed with p-P38 MAPK and P38 MAPK (Cell Signaling Technology) and detected using a secondary antibody (Epitomics) conjugated with horseradish peroxidase. Chemiluminescence was used to identify specific proteins according to the enhanced chemiluminescence (ECL) system.

### Statistical analysis

Comparisons between two groups were performed using Student’s *t*-test. Multiple group comparisons were performed using an analysis of variance (ANOVA). Differences were considered to be statistically significant at *p* < 0.05. All of the analyses were performed with the GraphPad Prism program (GraphPad Software, Inc. San Diego, CA).

## Results

### The establishment of an iron overload mouse model

**Figure 1:**
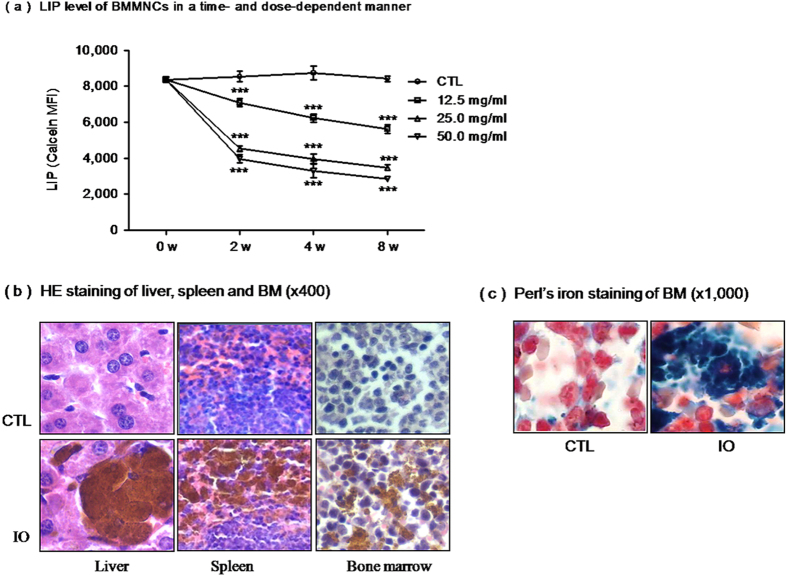
The establishment of an iron overload mouse model. ****** (**a**) Iron overload induced labile iron pool level (LIP) of bone marrow mononuclear cells (BMMNCs) in a time- and dose-dependent manner showing the mean fluorescent intensity (MFI) of calcein. (N = 3, ^***^P < 0.001 vs. CTL). (**b**) The hepatic, splenetic and bone marrow (BM) iron deposits were exposed to hematoxylin-eosin staining (x400). (**c**) BM cells were subjected to Perl’s iron staining (x1,000).

In a preliminary study, an iron overload mouse model was established by intraperitoneally injecting with different doses (12.5, 25 or 50 mg/ml) of iron dextran every three days for various durations (2 w, 4 w, 8 w). Comprehensively, we chose to intraperitoneally inject mice with 25 mg/ml iron dextran for 4 weeks as experimental conditions. To confirm the establishment of the iron overload mouse model, the labile iron pool (LIPs) of the BMMNCs were dynamically detected, while the hepatic, splenic and bone marrow (BM) iron deposits were assessed at the fourth week. Results showed that the LIP level of the BMMNCs was gradually increased in a time- and dose-dependent manner (Fig. 1a). Furthermore, iron deposits in the liver, spleen and BM cells were obviously observed after injecting mice with 25 mg/ml iron dextran for 4 weeks (Fig. 1b,c).

### Iron overload affected the ratio of immature hematopoietic cells

**Figure 2:**
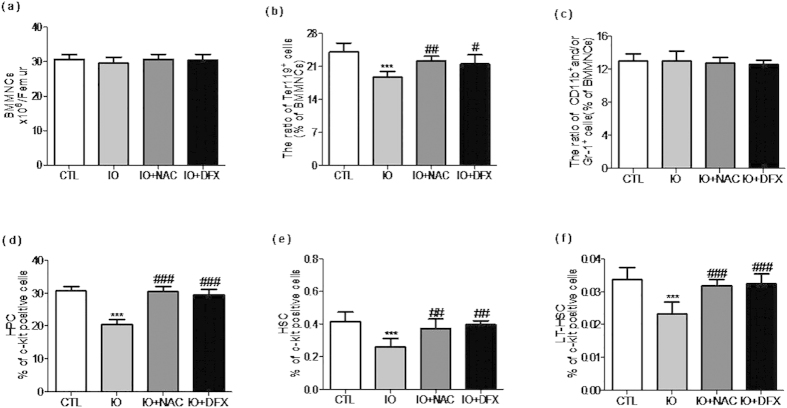
Iron overload selectively affected the frequencies of immature hematopoietic cells. ******Mice were injected with 25 mg/ml iron dextran in the iron overload group or were injected with normal saline in the control group. (**a**) The number of BMMNCs is presentedas the means ± SE of three independent experiments. (**b**–**c**) The frequencies of erythroid and myeloid cells are presented as the means ± SE of three independent experiments. (**d**–**f**) The frequencies of hematopoietic progenitor cells (HPCs) (Lin^-^c-kit^+^Sca^–^1^–^, LKS^–^), hematopoietic stem cells (HSCs) (Lin^–^c-kit^+^Sca^–^1^+^, LKS^+^) and long^–^term hematopoietic stem cells (LT-HSCs) (CD34^–^LKS^+^) in BMMNCs are presented as the means ± SE of three independent experiments. N = 5 mice/ group. ***P < 0.001 vs. CTL; ^*#*^*P* < 0.05, ^##^*P* < 0.01, ^*#*##^*P* < 0.001 vs. IO.

To determine whether iron overload affected bone marrow hematopoiesis, the ratio of immature hematopoietic cells in BM was analyzed. In the iron-overloaded BM, the percentage of the erythroid cells was significantly lower. However, there were no differences with the number of BMMNCs and the ratio of the myeloid cells(Fig. 2a–c). Compared with the control group, the percentage of all HPCs (Lin^–^c-kit^+^Sca-1^−^, LKS^−^), HSCs (Lin^–^c-kit^+^Sca-1, LKS^+^) and LT-HSCs (CD34^–^, LKS^+^) were significantly reduced(Fig. 2d–f). These findings demonstrate that iron overload selectively affected the ratio of immature hematopoietic cells.

### Iron overload injured the clonogenic capacity of HSPCs

**Figure 3:**
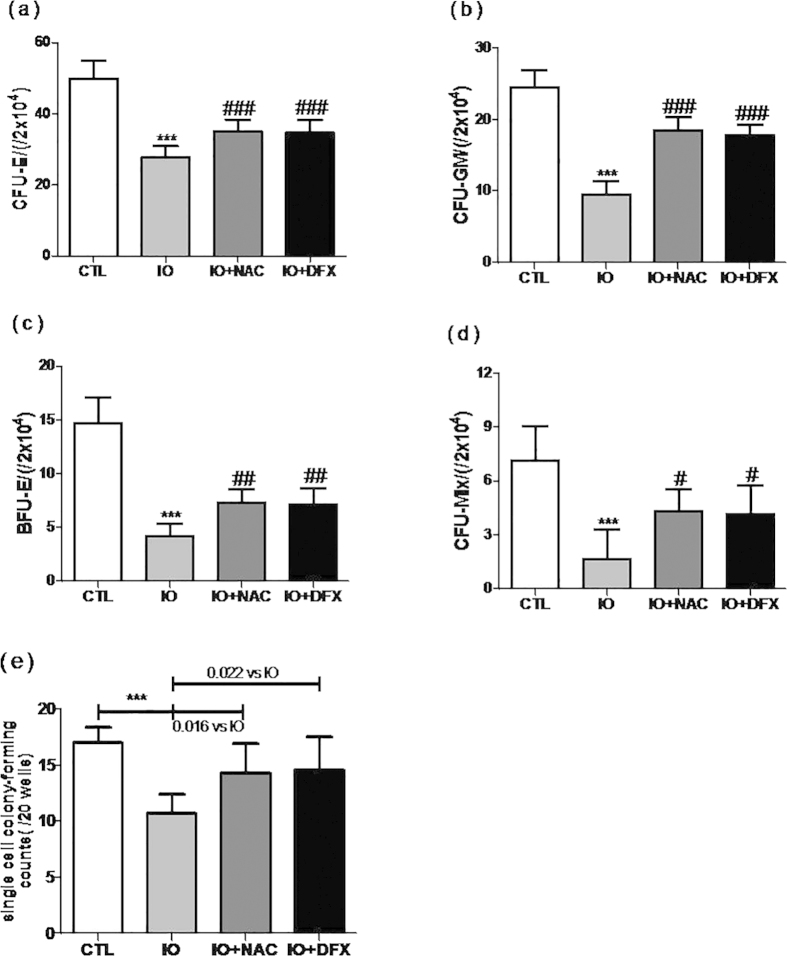
Iron overload damaged the clonogenic capacity of HSPCs. (a–d) Colony- forming cell (CFC) assays (CFU-E, BFU-E, CFU-GM and CFU-mix) were performed to determine the colony-forming viability of the HPCs. (**e**)Single cell colony-forming counts of the sorted HSCs were performed to determine the clonogenic capacity of the HSCs. The data are expressed as the means ± SE of three independent experiments. N = 6. ***P < 0.001 vs. CTL; ^*#*^*P* < 0.05, ^##^*P* < 0.01, ^###^*P* < 0.001. vs. IO.

We examined whether iron overload could have an effect on the clonogenic function of HSPCs. CFC assays were performed to determine the colony-forming viability of HPCs. The data revealed that hematopoietic colony-forming counts (CFU-E, BFU-E, CFU-GM and CFU-mix) in the iron overload group were markedly lower than thosein the control group (Fig. 3a–d). And the single cell colony-forming counts of the sorted HSCs were significantly reduced in iron-overloaded ones. (10.67 ± 0.72 vs. 17.00 ± 0.58) (Fig. 3e). Importantly, this effect could be reversed after treating iron-overloaded mice with DFX or NAC.

**Figure 4:**
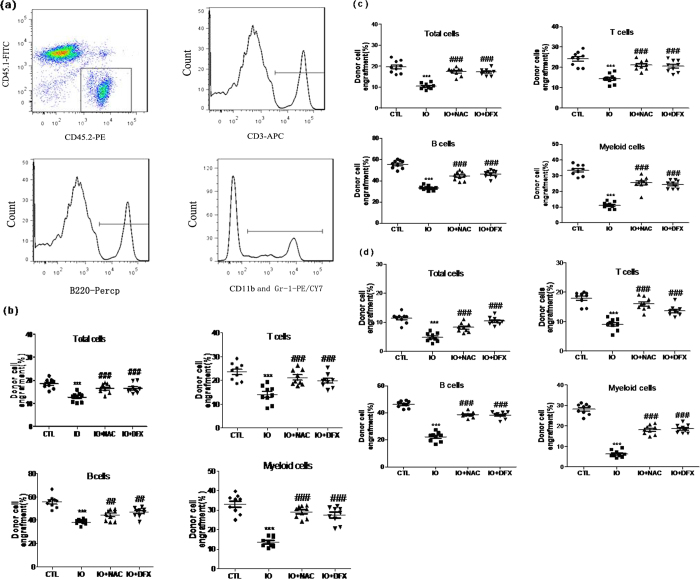
Iron overload impaired the function of HSC long-term and multi-lineage engraftment after bone marrow transplantation. Donor BMMNCs from iron- overloaded mice or normal mice (Ly45.2) were mixed with competitive cells and then transplanted into recipient mice (Ly45.1). (**a**) A representative flow cytometric analysis of peripheral blood cells from the recipient mice two months after transplantation is shown. (**b**) Donor cell-derived engraftment in the peripheral blood was measured 2 months after transplantation. (**c**) Donor cell-derived engraftment in the peripheral blood was measured 4 months after transplantation. (**d**) Donor cell-derived engraftment in the peripheral blood was measured after secondary transplantation. The data are expressed as the means ± SE of the percentage of donor-derived leukocytes (CD45.2^+^cells), T cells (CD45.2^+^CD3^+^cells), B cells (CD45.2^+^B220^+^cells), and myeloid cells (CD45.2^+^CD11b^+^and/or Gr-1^+^ granulocyte^-^monocyte-macrophage). N = 9 recipient mice/ group. ***P < 0.001 vs. CTL.

Long-term and multi-lineage engraftment is the gold standard assay to measure the self-renewal potential of HSCs. Therefore, whether long-term hematopoiesis was affected by iron overload was validatedby the application of competitive repopulation assays. Donor cell-derived engraftment, originating from peripheral blood cells of the recipient mice, was analyzed at the end of the 2nd and the forth months of BM transplantation. Immunophenotypes by using flow cytometry demonstrated that the recipient mice with iron-overloaded donors had lower levels of myeloid, B- and T-lymphocytic lineage engraftments compared that with the control donors (Fig. 4a). As shown in Fig. 4b, donor cell engraftment had a 1.46-fold decrease at the end of the 2nd month, and in the recipient mice the levels of myeloid, B- and T-lymphocytic lineage engraftments shown a 2.41-fold, 1.45-fold and 1.68-fold decrease, respectively. Similarly, donor cell engraftment decreased to different degreesat the end of the forth month (Fig. 4c). Furthermore, data of secondary transplantation demonstrated that iron overload could suppress long-term and multi-lineage hematopoiesis (Fig. 4d), further inducing bone marrow impairment. However, this effect could be reversed after administering the iron-overloaded mice with DFX or NAC.

### Iron overload selectively affected the hematopoietic system

Whether iron overload has an effect onthe hematopoietic systemwere determined by peripheral blood cells counts. The number of WBCs in iron-overloaded mice was significantly increased, whereas the proportion of neutrophils was markedly decreased (Table 1). The platelet count was also decreased, while the HGB level did not show a significant difference compared with the control group.

**Figure 5:**
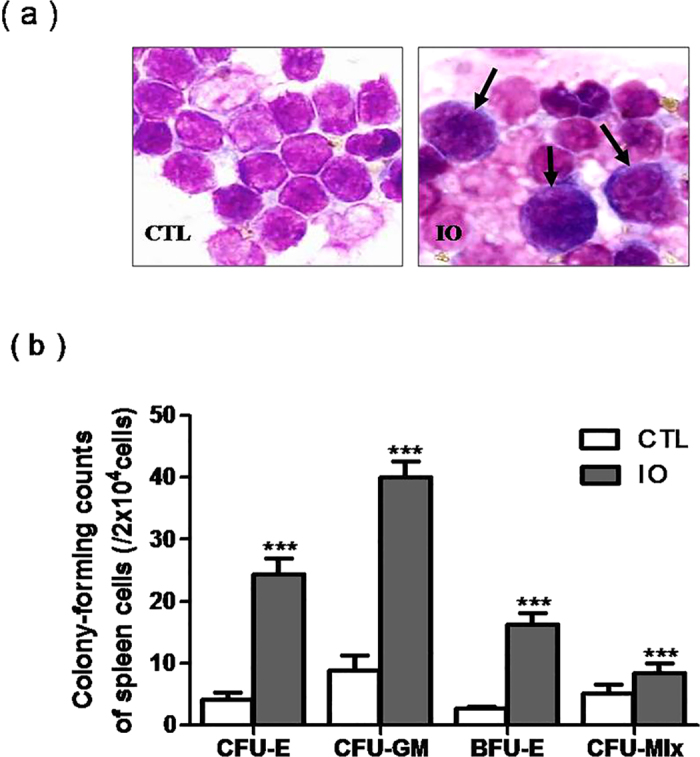
Iron overload led to extramedullary hematopoiesis. (**a**) Spleen cells were exposed to Wright's staining (x 1,000). (**b**) Hematopoietic colony-forming counts (CFU-E, BFU-E, CFU-GM and CFU-mix) from spleen cells are shown as the means ± SE of three independent experiments. N = 6. ***P < 0.001 vs. CTL.

In cases of bone marrow failure (caused by high-dose chemotherapy and/or total body irradiation or MDS), hematopoiesis is established in extramedullary sites, prominently in the spleen^12^. In the iron-overloaded mice, hematopoietic cells were observed in the smear aspirateof spleen cells (Fig. 5a). Spleen cells from normal and iron-overloaded mice were grown in M3434 semi-solid media, and the hematopoietic colony-forming counts (CFU-E, BFU-E, CFU-GM and CFU-mix) in the iron overload group were markedly increased (Fig. 5b,c). These results indicated that iron overload led to extramedullary hematopoiesis.

### Iron overload increased ROS production in BMMNCs and HSPCs

**Figure 6:**
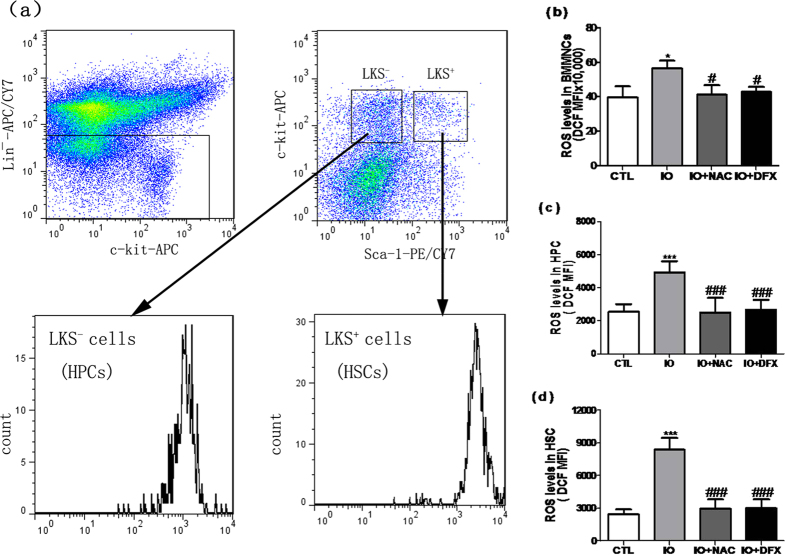
Iron overload enhanced intracellular ROS production. (**a**)A representative flow cytometric ROS analysis using DCF staining in HSCs and HPCs is shown. (**b**–**d**) The ROS levels in the BMMNCs, HPCs and HSCs are presented as the means ± SE of the DCF MFI from three independent experiments. N = 5 mice/group. ^*^P < 0.05, ***P < 0.001 vs. CTL; ^*#*^*P* < 0.05, ^###^*P* < 0.001. vs. IO.

Based on literatures and our preliminary study, iron overload could negatively affect vital organs, such as the liver, heart, and endocrine glands, via producing ROS^3^,^4^,^5^,^13^. In this study, we investigated the levels of ROS in iron-overloaded mice. The levels of ROS by the analysis of a representative flow cytometry revealed a 1.43-fold, 1.94-fold and 3.48-fold increase in BMMNCs, HPCs and HSCs, respectively. (Fig. 6a–d). Moreover, DFX or NAC treatment significantly inhibited the ROS which was induced by iron overload in hematopoietic cells.

### Iron overload activated the NOX4/ROS/P38 MAPK signaling pathways.

**Figure 7:**
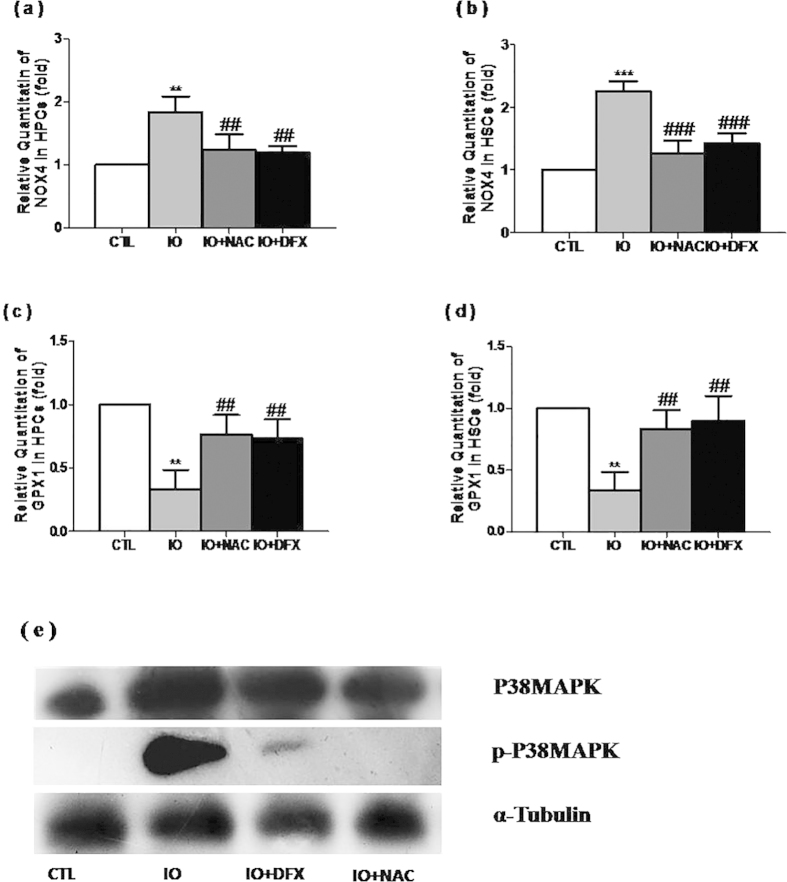
Iron overload activated the NOX4/ROS/P38MAPK signaling pathways.(a–d) The levels of NOX4 and GPX1 mRNA expression are expressed as the means ± SE of fold changes compared with their respective controls. N = 3, ^**^P < 0.05, ***P < 0.001 vs. CTL; ^*##*^*P* < 0.05, ^###^*P* < 0.001. vs. IO. (**e**) The expression of p-P38 MAPK and P38 MAPK were analyzed via Western blotting. α-tubulin was used as a loading control.

Iron overload upregulated the expression of NOX4 mRNA and downregulated the expression of GPX1 mRNA in the HSPCs to variable degrees (Fig. 7a–d). Furthermore, the protein levels of both P38 MAPK and p-P38 MAPK were significantly increased after ironoverload (Fig. 7e). Contrarily, DFX or NAC treatment reduced the level of these proteins to a different degree. These results suggested that iron overload induced chronic oxidative stress in HSPCs via the NOX4/ROS/P38 MAPK signaling pathways to some extent.

## Discussions

Iron overload is a disorder of iron metabolism that could be induced by multiple blood transfusions and excess gastrointestinal absorption. The disorder leads to tissue damage and ultimately to the dysfunction of visceral organs. Recently, numerous reports have stated that iron overload impairs the proliferation of erythroid progenitor cells from patients with MDS^14^,^15^. Iron chelation therapy has been reported to enhance erythropoiesis and reduce the cytopenia in patients with iron-overload anemia^16^,^17^. In this study, we established an iron overload mouse model and explored effects of iron overload on the hematopoietic system. The results showed that iron overload affected the functions of HSPCs and reduced the number of HSCs. Moreover, the hematopoietic inhibitory effects were closely related to oxidative stress.

Numerous reports have demonstrated that iron overload has negative effects on parenchymal organs. Sampaio AF *et al.* found that iron toxicity enhanced tissue damage by the mediation of oxidative stress in an animal model of diabetes^18^. It was also reported that injecting mice with increased skeletal muscle iron content induced oxidative stress and reduced exercise performance^19^. Thus, it is necessary to establish an iron-overload model to study changes in the hematopoietic system. In our preliminary studies, *in vitro* iron-overloaded BMMNCs and MSCs were successfully established *in vitro* and showed the inhibitory effects on bone marrow^8^. In this study, an iron-overloaded mouse model, showing the inhibitory effects caused by iron overload, was established. To dynamically monitor the conditions of iron load, mice were regularly injected with different doses of iron dextran over varying periods of weeks. In these mice, the LIP level of the BMMNCs was significantly increased. On the basis of comprehensively physical conditions and the mental state, injecting mice with 25 mg/ml iron dextran for 4 weeks was used for establishing the iron overload model, and iron deposits presented in the smear aspirate ofliver, spleen and BM cells. These results further validated the iron-overloaded mouse model.

The hematopoietic system includes HSCs and HPCs. During differentiation, the progeny of HSCs proceed through a series of lineage commitment steps, progressively losing their self-renewal potential and becoming more restricted in their differentiation capacity^20^,^21^,^22^,^23^. In this study, we explored whether iron overload could cause injury of hematopoietic stem progenitor cells. The CFC results revealed that iron overload led to the decrease of hematopoietic colony-forming units. Iron overload also damaged the function of the HPCs. To observe HSC function, we performed a single cell clone culture and a competitive transplantation experiment. The results showed that iron overload significantly weakened the clonogenicity and the capacity of hematopoietic reconstitution in HSCs. These findings demonstrate that iron overload induces acute bone marrow damage and causes long-term BM injury.

The WBC count was significantly increased in the iron-overloaded mice, whereas there was no obvious change in HGB. These results are not consistent with clinical data or total body irradiation data^24^,^25^,^26^,^27^. One reason may be that bone marrow normally has robust hematopoietic reserves. Although iron overload damages HSC function, it is not sufficient to induce a significant change in the hematopoietic progenitor cell count of peripheral blood. The other reason could be extramedullary hematopoiesis, which was validated by the observation of hematopoietic cells in the smear aspirate of spleen cells and by CFU-GEMM. These results indicate that the diseases caused by iron overload occur gradually. As a result, we should pay attention to clinical prevention.

Normally, ROS are formed by internal oxygen metabolites, including polar molecules (such as O2 -, OH, HO2, RO2, RO, etc.) and a nonpolar molecule (such as H2O2 and O2). ROS could be produced in a variety of ways, including the NADPH oxidase pathway and the mitochondrial electron transport chain. Keeping ROS at an appropriate level plays an important role in some biological phenomena, such as the activation of signaling pathways and the signals involved in gene expression. However, increasing ROS and (or) damage of antioxidant systemcould lead to oxidative stress reaction. ROS could also trigger a variety of signal transduction pathways, such as p38 MAPK/p53 and p21 WAF1/Cip1/Sdi1, eventually leading to different reactions, such as cell proliferation differentiation and apoptosis, that cause tissue, organ and cell damage. A large number of literatures and early studies demonstrated that iron overload led to an increase in cellular ROS and negatively affected vital organs, such as the liver, heart, and endocrine glands^3^,^4^,^5^,^28^. In this experiment, iron overload led to the increase of ROS levels, too. It is consistent with the results of other *in vitro* studies^8^. An increasing amount of evidence indicates that cells could actively produce ROS through a family of tightly regulated NADPH oxidases (NOXs), homologues of phagocyte oxidase^29^,^30^,^31^. In this study, iron overload was found to upregulate the expression of NOX4 in HPCs and HSCs. Therefore, NOX4 might play a role in the production of ROS. P38 belongs to the MAPK family of signal transduction kinases^32^. It regulates a variety of cellular processes, such as inflammation, cell cycle arrest, and apoptosis. It also plays a critical role in the DNA damage resulting from genotoxic and oxidative stress. In this study, the protein levels of both p-p38 MAPK and p38 MAPK exhibited a significant increase after ironoverload. To some extent, these results confirmed that iron overload might damage hematopoiesis via the NOX4-ROS-P38 MAPK signaling pathway. Interestingly, these effects were abrogated by treating with NAC or DFX, suggesting that iron overload could be closely related to oxidative stress.

Hiroshi Okabe *et al.* recently reported that the bone marrow hematopoietic microenvironment was impaired in iron-overloaded mice, whereas bone marrow transplantation did not show any significant defects in the capacity of hematopoietic reconstitution in HSCs of iron-overloaded mice, i.e., HSCs were not significantly affected by excess iron. These results contradict our observations. The capacity of hematopoietic reconstitution in hematopoietic stem/progenitor cells of in iron-overloaded mice could be significantly affected by excess iron if to observe a longer period of time. Similarly, our animal models might not reflect the precise pathological condition of iron-overload patients, and other mice models might be necessary to understand how excess iron affects hematopoiesis^33^.

An iron overload model was successfully established by injecting with iron dextran and was able to stimulate clinical situations. Further study indicated that iron overload impaired the hematopoiesis of bone marrow. However, iron overload could induce extramedullary hematopoiesis during the early period of time. This research provided an experimental model that could be used for further study on the mechanism of iron overload diseases. It is also useful for finding new therapeutic targets inthe treatment of iron overloaded patients with hematopoietic dysfunction.

## Author Contributions

M.Z. and A.M. designed the research and helped perform the analysis with constructive discussions. X.C. and D.L. carried out the experiments, analyzed the data, interpreted the results and prepared the manuscript. X.C. and Y.Z. raise animals and carried out the experiments. J.M., W.L., X.X., C.L., J.M., J.C. and Q.L. contributed to data collection and interpretation. J.W. helped perform the analysis with constructive discussions.

## Additional Information

**How to cite this article**: Chai, X. *et al.* ROS-mediated iron overload injures the hematopoiesis of bone marrow by damaging hematopoietic stem/progenitor cells in mice. *Sci. Rep.* **5**, 10181; doi: 10.1038/srep10181 (2015).

## Figures and Tables

**Table 1:** Counts of peripheral blood cells (x ± s, N = 5).

**Groups**	**WBC (x10**^**9**^**/L)**	**NE (%)**	**LY (%)**	**HGB (g/L)**	**PLT (x10**^**9**^**/L)**
**CTL**	9.6 ± 0.4	13.3 ± 1.1	86.9 ± 1.2	122.8 ± 4.8	543.6 ± 19.6
**IO**	12.2 ± 0.6^a^	9.1 ± 1.2^a^	91.4 ± 1.0^a^	107.6 ± 3.7	436.6 ± 11.4^a^
**IO+DFX**	9.7 ± 0.9^b^	12.9 ± 0.3^b^	86.2 ± 0.7^b^	117.4 ± 4.1	463.0 ± 16.2^b^
**IO+NAC**	8.2 ± 0.6^b^	11.6 ± 1.1^b^	85.9 ± 0.5^b^	108.5 ± 2.5	465.8 ± 8.5^b^

WBC: White blood cell; NE: Neutrophil; LY: Lymphocyte; HGB: Hemoglobin; PLT: Platelet

## References

[b1] Suzuki T. *et al.* Japanese epidemiological survey with consensus statement on Japanese guidelines for treatment of iron overload in bone marrow failure syndromes. Int. J. Hematol. 88, 30–35 (2008). 18581199 10.1007/s12185-008-0119-y PMC2516546

[b2] Bird R. J. *et al.* When should iron chelation therapy be considered in patients with myelodysplasia and other bone marrow failure syndromes with iron overload? Intern Med. J. 42, 450–455(2012). 22498118 10.1111/j.1445-5994.2012.02734.x

[b3] Danjou F. *et al.* Longitudinal analysis of heart and liver iron in thalassemia major patients according to chelation treatment. Blood Cells Mol. Dis. 51, 142–145 (2013). 23816436 10.1016/j.bcmd.2013.06.001

[b4] Sampaio A. F. *et al.* Ir n toxicity mediated by oxidative stress enhances tissue damage in an animal model of diabetes. Biometals 27, 349–361 (2014). 24549594 10.1007/s10534-014-9717-8

[b5] Piloni N. E. *et al.* Acute iron overload and oxidative stress in brain. Toxicology. 314, 174–182 (2013). 24120471 10.1016/j.tox.2013.09.015

[b6] Pullarkat V. Objectives of iron chelation therapy in myelodysplastic syndromes: more than meets the eye? Blood. 114, 5251 –5255 (2009). 19710499 10.1182/blood-2009-07-234062

[b7] Lee S. E. *et al.* Improvement in hematopoiesis after iron chelation therapy with deferasirox in patients with aplastic anemia. Acta. Haematol 129, 72–77 (2013). 23154600 10.1159/000342772

[b8] Lu W. *et al.* Free iron catalyzes oxidative damage to hematopoietic cells/mesenchymal stem cells *in vitro* and suppresses hematopoiesis in iron overload patients. Eur. J. Haematol 91, 249–261 (2013). 23772810 10.1111/ejh.12159

[b9] Meng A. *et al.* Ionizing radiation and busulfan inhibit murine bone marrow cell hematopoietic function via apoptosis-dependent and -independent mechanisms. Exp. Hematol 31, 1348–1356 (2003). 14662344 10.1016/j.exphem.2003.08.014

[b10] Zhang H. *et al.* Resveratrol ameliorates ionizing irradiation-induced long-term hematopoietic stem cell injury in mice. Free Radic. Biol. Med. 54, 40–50 (2013). 23124026 10.1016/j.freeradbiomed.2012.10.530 PMC4711372

[b11] Meng A. *et al.* Ionizing radiation and busulfan induce premature senescence in murine bone marrow hematopoietic cells. Cancer Res. 63, 5414–5419 (2003). 14500376

[b12] Horváth Z. *et al.* Extramedullary hematopoiesis is dysregulated in histamine-free histidine decarboxylase knockout (HDC^-/-^) mice. Inflamm. Res. 59, 429–436 (2010). 19921486 10.1007/s00011-009-0114-7

[b13] Camaschella C. Understanding iron homeostasis through genetic analysis of hemochromatosis and related disorders. Blood. 106, 3710–3717 (2005). 16030190 10.1182/blood-2005-05-1857

[b14] Hartmann J. *et al.* Iron overload impairs proliferation of erythroid progenitors cells (BFU-E) from patients with myelodysplastic syndromes. Leuk. Res. 37, 327–332 (2013). 23259989 10.1016/j.leukres.2012.11.005

[b15] Taoka K. *et al.* The effect of iron overload and chelation on erythroid differentiation. Int. J. Hematol. 95, 149–159 (2012). 22193844 10.1007/s12185-011-0988-3

[b16] Oliva E. N. *et al.* Iron chelation therapy associated with improvement of hematopoiesis in transfusion-dependent patients. Transfusion 50, 1568–1570 (2010). 20230535 10.1111/j.1537-2995.2010.02617.x

[b17] Heli H., Mirtorabi S. & Karimian K. Advances in iron chelation: an update. Expert Opin. Ther. Pat. 21, 819–856 (2011). 21449664 10.1517/13543776.2011.569493

[b18] Sampaio A. F. *et al.* Iron toxicity mediated by oxidative stress enhances tissue damage in an animal model of diabetes. Biometals 27, 349–361 (2014). 24549594 10.1007/s10534-014-9717-8

[b19] Reardon T. F. & Allen D. G. Iron injections in mice increase skeletal muscle iron content, induce oxidative stress and reduce exercise performance. Exp. Physiol. 94, 720–730 (2009). 19201785 10.1113/expphysiol.2008.046045

[b20] Juntilla M. M. *et al.* AKT1 and AKT2 maintain hematopoietic stem cell function by regulating reactive oxygen species. Blood. 115, 4030–4038 (2010). 20354168 10.1182/blood-2009-09-241000 PMC2875090

[b21] Yamane T., Washino A. & Yamazaki H. Common developmental pathway for primitive erythrocytes and multipotent hematopoietic progenitors in early mouse development. Stem Cell Reports 1, 590–603 (2013). 24371812 10.1016/j.stemcr.2013.10.008 PMC3871389

[b22] Miyamoto T. Role of osteoclasts in regulating hematopoietic stem and progenitor cells. World. J. Orthop. 4, 198–206 (2013). 24147255 10.5312/wjo.v4.i4.198 PMC3801239

[b23] Martinez-Agosto J. A., Mikkola H. K., Hartenstein V. & Banerjee U. The hematopoietic stem cell and its niche: a comparative view. Genes Dev. 21, 3044–3060 (2007). 18056420 10.1101/gad.1602607

[b24] Kikuchi S, Kobune M, Iyama S, *et al.* Improvement of iron-mediated oxidative DNA damage in patients with transfusion-dependent myelodysplastic syndrome by treatment with deferasirox. Free Radic. Biol. Med. 53, 643–648 (2012). 22705364 10.1016/j.freeradbiomed.2012.06.006

[b25] Rojas S. M. *et al.* Transfusion dependence development and disease evolution in patients with MDS and del(5q) and without transfusion needs at diagnosis. Leuk. Res. 38, 304–309 (2014). 24333115 10.1016/j.leukres.2013.11.005

[b26] Wang Y. *et al.* Total body irradiation causes residual bone marrow injury by induction of persistent oxidative stress in murine hematopoietic stem cells. Free Radic. Biol. Med. 48, 348–356 (2010). 19925862 10.1016/j.freeradbiomed.2009.11.005 PMC2818724

[b27] Shao L. *et al.* Total body irradiation causes long-term mouse BM injury via induction of HSC premature senescence in an Ink4a- and Arf-independent manner. Blood. 123, 3105–3115 (2014). 24622326 10.1182/blood-2013-07-515619 PMC4023419

[b28] Andrews N. C. Disorders of iron metabolism. N. Engl. J. Med. 341, 1986–1995 (1999). 10607817 10.1056/NEJM199912233412607

[b29] Lambeth J. D. Nox enzymes, ROS, and chronic disease: an example of antagonistic pleiotropy. Free Radic. Biol. Med. 43, 332–347 (2007). 17602948 10.1016/j.freeradbiomed.2007.03.027 PMC2013737

[b30] Bedard K. & Krause K. H. The NOX family of ROS-generating NADPH oxidases: physiology and pathophysiology. Physiol. Rev. 87, 245–313 (2007). 17237347 10.1152/physrev.00044.2005

[b31] Shao L. *et al.* Reactive oxygen species and hematopoietic stem cell senescence. Int. J. Hematol. 94, 24–32 (2011). 21567162 10.1007/s12185-011-0872-1 PMC3390185

[b32] Kyriakis J. M. & Avruch J. Mammalian mitogen-activated protein kinase signal transduction pathways activated by stress and inflammation. Physiol. Rev. 81, 807–869 (2001). 11274345 10.1152/physrev.2001.81.2.807

[b33] Okabe H. *et al.* The bone marrow hematopoietic microenvironment is impaired in iron-overloaded mice. Eur. J. Haematol 93, 118–128 (2014). 24628561 10.1111/ejh.12309

