# Corrigendum: ROS-mediated iron overload injures the hematopoiesis of bone marrow by damaging hematopoietic stem/progenitor cells in mice

**DOI:** 10.1038/srep41900

**Published:** 2017-02-09

**Authors:** Xiao Chai, Deguan Li, Xiaoli Cao, Yuchen Zhang, Juan Mu, Wenyi Lu, Xia Xiao, Chengcheng Li, Juanxia Meng, Jie Chen, Qing Li, Jishi Wang, Aimin Meng, Mingfeng Zhao

Scientific Reports 5: Article number: 10181110.1038/srep10181; published online: 05 13 2015; updated: 02 09 2017

The iron-overload mouse model used in this study was previously reported by the authors in references 1 and 2, which are not cited in the Article.

The data supporting the mouse model is presented in Figure 1 of the Article. The data presented in in Figure 1b appear in reference 1 as Figure 1b; and the data presented in Figure 1c appear in reference 1 as Figure 1c and in reference 2 as Figure 1.

Therefore the legend of Figure 1 should read:

**Figure 1.** **The establishment of an iron overload mouse model.** (**a**) Iron overload induced labile iron pool level (LIP) of bone marrow mononuclear cells (BMMNCs) in a time- and dose-dependent manner showing the mean fluorescent intensity (MFI) of calcein. (N = 3, ^***^P < 0.001 vs. CTL). (**b**) The hepatic, splenetic and bone marrow (BM) iron deposits were exposed to hematoxylin-eosin staining (x400). Republished from [1]. (**c**) BM cells were subjected to Perl’s iron staining (x1,000). Republished from [1], and from [2] with permission.

The authors apologize for the omission of this information in the original Article.
